# Glucose Metabolic Reprogramming in Colorectal Cancer: From Mechanisms to Targeted Therapy Approaches

**DOI:** 10.1002/cam4.71185

**Published:** 2025-08-30

**Authors:** Runkai Zhou, Fazhi Wang, Jiazhe Wen, Xuefeng Zhou, Yugang Wen

**Affiliations:** ^1^ Department of General Surgery Shanghai General Hospital, Shanghai Jiao Tong University School of Medicine Shanghai China; ^2^ School of Basic Medical Sciences, Fudan University Shanghai China; ^3^ Department of Oncology The Dongtai Hospital of Nantong University Dongtai Jiangsu China

**Keywords:** colorectal cancer, glucose metabolism, metabolic reprogramming, targeted therapy

## Abstract

**Background:**

Colorectal cancer (CRC) is one of the most common malignant tumors, and its morbidity ranks third among all cancers, with a trend toward younger patients. Metabolic reprogramming, a unique metabolic mode in tumor cells, is closely related to the occurrence and development of CRC. Numerous studies have confirmed that many genetic and protein changes can regulate cellular metabolic reprogramming, of which changes in glucose metabolism have the greatest impact. These aberrant metabolic processes provide energy and essential nutrients to CRC cells, promoting their proliferation and metastasis and influencing tumor resistance. The purpose of this review is to outline the role of glucose metabolic reprogramming in the onset and development of CRC, discuss the research progress in the dual reprogramming of glucose metabolism and lipid metabolism or glucose metabolism and amino acid metabolism, and address the issues of targeted metabolism therapy and drug resistance.

**Methods:**

We searched PubMed for review articles published in English between January 1, 2015, and April 26, 2024, which included “Colorectal Neoplasms” with “Metabolic Reprogramming” OR “Glucose Metabolism Disorders” OR “The Warburg Effect” OR “Targeted Therapy.” Subsequently, the literature was classified, organized, and summarized. Various types of studies were integrated and compiled into this review. Additionally, mechanism diagrams were drawn to facilitate the understanding of this study. The figures were created using BioRender.com and has obtained the official publication license.

**Conclusions:**

Glucose metabolic reprogramming serves as a pivotal driver of CRC initiation, progression, and chemoresistance, while its crosstalk with lipid or amino acid metabolic reprogramming further amplifies the malignant phenotype of CRC. Targeted therapeutic strategies aiming at glucose metabolic reprogramming (such as metabolic inhibitors, combination with immunotherapy) and related clinical research have demonstrated potential for inhibiting CRC progression and improving treatment outcomes.

**Abbreviations**
2‐DG: 2‐Deoxy‐D‐glucose
4‐OI: 4‐Octyl itaconate
ADMA: asymmetric dimethylarginine
AF9: ALL1‐fused gene from chromosome 9
AMPK: AMP‐activated protein kinase
ARL15: ADP‐ribosylation factor‐like 15
ART1: arginine ADP‐ribosyltransferase 1
ATF4: transcription factor 4
ChREBP: carbohydrate response element binding protein
CRC: colorectal cancer
CRMP2: collapsin response mediator protein 2
DC: dendritic cells
FABP4: fatty‐acid‐binding protein 4
FBP: fructose‐1,6‐bisphosphate
FOXM1: Forkhead box M1
G6PD: glucose‐6‐phosphate dehydrogenase
GAPDH: glyceraldehyde‐3‐phosphate dehydrogenase
GLUT: glucose transporter
GPR37: G protein‐coupled receptor 37
GSK‐3: glycogen synthase kinase‐3
HIF1‐α: hypoxia‐inducible factor 1 alpha
HK2: hexokinase 2
HKDC1: hexokinase domain component 1
LDH: lactate dehydrogenase
MnSOD: manganese superoxide dismutase
OXPHOS: oxidative phosphorylation
PBX3: Pre‐B‐cell leukemia transcription factor 3
PCK: phosphoenolpyruvate carboxykinase
PEPCK: phosphoenolpyruvate carboxykinase
PFKFB3: fructose‐2,6‐biophosphatase 3
PGC‐1α: peroxisome proliferator‐activated receptor gamma coactivator‐1α
PGD: phosphogluconate dehydrogenase
PHD2: prolyl hydroxylase domain protein 2
PLOD2: procollagen‐lysine, 2‐oxoglutarate 5‐dioxygenase 2
PROX1: prospero‐related homeobox 1
RARRES1: retinoic acid receptor responder 1
ROS: reactive oxygen species
RPIA: ribose‐5‐phosphate isomerase A
SCD1: stearoyl‐CoA desaturase 1
SELENBP1: selenium‐binding protein 1
SIRT1: silent information regulator of transcription, sirtuin 1
SNP: single nucleotide polymorphisms
SOX2: sex‐determining region Y‐box2
TCA cycle: tricarboxylic acid cycle
TME: tumor microenvironment
Trx‐1: thioredoxin‐1
XN: xanthohumol

## 1 Introduction

Colorectal cancer (CRC) is the third most common cancer and the second leading cause of cancer‐related death globally [1]. CRC is a multifactorial disease influenced by both environmental and genetic factors. In addition to aging, adverse factors such as obesity, physical inactivity, and smoking further increase the risk of developing CRC [2]. Between 2012 and 2021, owing to health education and the reduction in smoking, the mortality rate of CRC patients has declined by 1.8% per year in both men and women [3]. Despite continuous advancements in screening methods, with significant improvements in detection rates, the mortality of advanced CRC patients remains unacceptably high even after undergoing comprehensive treatments such as surgery, radiation therapy, chemotherapy, targeted therapy, and immunotherapy [4].

Metabolic reprogramming is a hallmark of cancer cells, representing a core characteristic of malignancy wherein alterations in the quantity and properties of metabolic enzymes, upstream regulatory molecules, and downstream metabolic products occur [5]. To meet the heightened demand for energy and nutrients, tumor cells have developed novel metabolic pathways involving modifications in the glycolytic pathway, induction of the Warburg effect, lipid metabolism remodeling, regulation of amino acid metabolism, alterations in one‐carbon unit metabolism, and nucleotide metabolism [6]. Among these, glucose metabolic reprogramming has been proven to occur in various tumors, playing crucial roles in gene transcription, DNA damage repair, and the regulation of epigenetic modifications [7, 8, 9].

The primary goal of glucose metabolic reprogramming in CRC cells is to maximize anabolic metabolism and obtain the energy required for rapid growth. Other functions include providing anti‐apoptotic signals and substances that allow cancer cells to evade programmed cell death, thereby increasing their survival and proliferation. Additionally, considering that cancer cells often experience hypoxic conditions, glucose metabolic reprogramming assists in their adaptation and survival in low‐oxygen environments [10]. These aberrant metabolic processes provide energy and essential nutrients for CRC cells, thereby promoting their proliferation and metastasis [11]. Moreover, aberrant metabolism often leads to reduced chemotherapy efficacy, thus increasing the complexity of treatment [12, 13].

In recent years, with the development of experimental techniques, the targeting of genes and proteins related to metabolic reprogramming has provided a new direction for the prevention and treatment of CRC [14, 15]. Its core principle is to selectively inhibit or modulate key metabolic enzymes or pathways on the basis of the characteristics of tumor cell metabolic reprogramming, thereby suppressing the proliferation of tumor cells. Detection technologies based on tumor metabolism (such as fluorodeoxyglucose (FDG)‐PET) have been widely applied in clinical trials of cancer [16]. These technologies can not only help identify the metabolic characteristics of tumor cells but also be used to evaluate the effects of targeted metabolism therapies.

Cellular metabolism is an integrated and dynamic process. The metabolism of the three major nutrients—glucose, lipids, and amino acids—is closely interconnected. This interconnection is further accentuated in the metabolic reprogramming of tumor cells [17]. Many factors that modulate the reprogramming of glucose metabolism in CRC also concurrently influence lipid or amino acid metabolism, thereby collectively impacting the biological characteristics of CRC. In this review, we analyze how glucose metabolic reprogramming in CRC interacts with other metabolic pathways, such as amino acid and lipid metabolism, to collectively impact tumor progression. Our review highlights the compilation and analysis of studies involving dual metabolic reprogramming related to glucose metabolism and lipid metabolism or glucose metabolism and amino acid metabolism. Additionally, we reviewed and integrated research on cuproptosis, a form of programmed cell death that has been widely studied in recent years, elucidating its underlying mechanisms and its relationship with glucose metabolic reprogramming. These two novel perspectives provide new insights into the future research directions for CRC.

This article systematically elaborates on glucose metabolic reprogramming in CRC, introducing potential mechanisms that affect tumor growth, proliferation, and metastasis. The impact of concurrent dual metabolic reprogramming in CRC was systematically analyzed. Targeted therapies, new drug developments, and clinical trials related to glucose metabolic reprogramming are also discussed. In summary, we described how glucose metabolism‐related molecules interact with carcinogenic factors and lead to the occurrence and development of CRC, which may help better understand their potential roles in intestinal diseases.

## 2 Mechanism of Glucose Metabolism Reprogramming in CRC

Glucose plays a pivotal role in cell metabolism as a primary energy source. It is involved in various metabolic processes, including glycolysis, the tricarboxylic acid cycle, the pentose phosphate pathway, gluconeogenesis, and glycogen metabolism. In addition to producing ATP, the energy currency of the cell, glucose can also be utilized in the synthesis of macromolecules such as nucleic acids, proteins, and lipids. Its metabolism also influences crucial cellular processes, including the regulation of gene expression and signaling pathways [18].

### 2.1 The Warburg Effect

The Warburg effect was the first formally proposed metabolic reprogramming pathway. In the 1920s, Otto Warburg discovered that cancer tissue slices consumed a substantial amount of glucose in vitro to produce lactate, even under aerobic conditions [19]. This process provides additional energy and nutrients to tumor cells. In 1929, the British biochemist Herbert Crabtree subsequently expanded upon Warburg's work and studied the heterogeneity of glycolysis in different tumor types. They confirmed the Warburg effect and further discovered that there are differences in the levels of respiration among different types of tumor cells, with many tumors exhibiting extensive respiration. To some extent, the carbohydrate metabolism of tumors is influenced by the environment in which they grow [20].

Owing to the limitations of the experimental conditions at the time, Warburg and colleagues were unable to investigate the mechanism behind the phenomenon, nor did they know what specifically caused it, whether it was driven by intrinsic factors or propelled by external factors. Warburg hypothesized that cancer is ultimately triggered by chemicals that destroy cellular respiratory organs, which are now known as mitochondria, damaging the ability of cells to perform oxidative phosphorylation (OXPHOS). Normal somatic cells meet their energy requirements by breathing oxygen, whereas cancer cells generate energy primarily through fermentation. Therefore, if the respiratory function of body cells remains intact, cancer can be prevented [21]. Racker further investigated and developed this theory by analyzing the differences in the respiratory capacity of tumor cells from the perspective of imbalanced intracellular pH and active‐defective ATP enzymes [22, 23]. Subsequent observations revealed that aerobic glycolysis is a controllable process that can be directly influenced by modulating growth factor signals, providing new insights into the development of the Warburg effect [24, 25].

**FIGURE 1:**
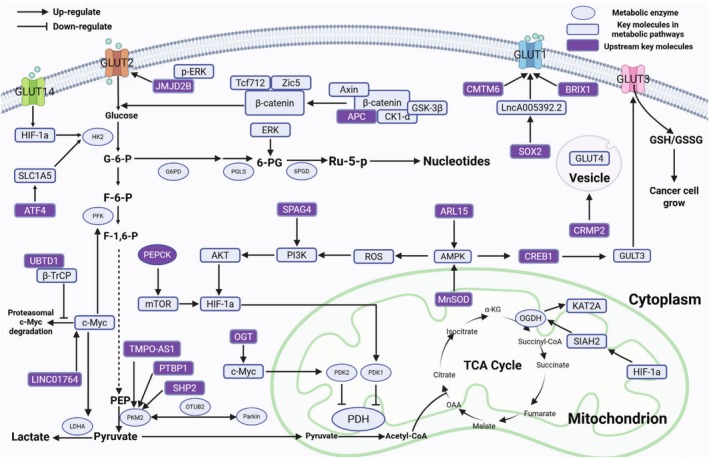
Illustration of the genes associated with metabolic reprogramming in the Warburg effect and the TCA cycle in CRC cells, along with their interconnections.

However, the causative theory proposed by Warburg was later proven incorrect. In 2006, Leder et al. demonstrated that knocking out lactate dehydrogenase (LDH) to inhibit aerobic glycolysis in cancer cells led to an oxygen‐dependent compensatory increase in oxygen consumption and OXPHOS. Knockout of LDH inhibits the proliferative capacity of cancer cells. Therefore, tumor cells retain the ability for normal respiration but preferentially utilize aerobic glycolysis to rapidly produce less ATP, enabling them to grow and divide more quickly [26]. With the development of genetics and metabolomics, oncogenes and tumor suppressor genes were subsequently discovered, demonstrating a causal relationship between their mutations and malignant transformation. The linkage between genes and metabolism offers new insights into the Warburg effect, thus ushering research on the Warburg effect into a new phase (Figure 1).

Over time, scientists discovered that the Warburg effect is evident in nearly all solid tumor cells, including CRC cells, and serves as an “energy supply station” for tumor growth and proliferation [27]. It also promotes the invasion and metastasis of CRC by reshaping the tumor microenvironment (TME). The Warburg effect enhances the ability of cancer cells to produce lactate, leading to acidification of the TME, and an acidic pH can accelerate the migration rate of tumor cells [28].

In recent years, on the basis of the metabolic characteristics of tumor cells and the expression levels of the Warburg effect, some scholars have classified tumors into different Warburg subtypes for research purposes [29, 30, 31]. A Dutch study used immunohistochemistry to quantify and analyze the expression levels of six proteins related to the Warburg effect (LDHA, GLUT1, MCT4, PKM2, p53, and PTEN) and classified CRC patients into three Warburg subtypes (low/medium/high) on the basis of these levels. The results revealed that patients with the high‐Warburg phenotype had the worst disease‐specific and overall survival rates. Nevertheless, there are many challenges in investigating the value of the Warburg subtype in the diagnosis and treatment of CRC patients [32]. A comprehensive multicenter study investigating the association between the Warburg effect and the TME revealed no significant correlation between the Warburg phenotype and the tumor‐infiltrating lymphocyte score or stromal score. The Warburg subtype cannot be directly linked to the quantity of lymphocytes infiltrating CRC or to the relative amount of tumor stroma content [33]. Furthermore, the Warburg subtype may also be correlated with tumor immune cells and cancer‐associated fibroblasts. This classification has promising implications for the metabolic treatment of patients with CRC. Personalized metabolic treatments can be implemented by discerning the metabolic characteristics of tumors in different patients. In summary, the influence of the Warburg effect on the treatment strategies and prognosis of CRC patients warrants further investigation [34].

The development of genomics and proteomics has also aided the study of the Warburg effect. On the basis of epigenetic studies of non‐coding RNAs, researchers have identified key factors in CRC cells associated with the Warburg effect, including transcription‐related factors (KRAS, APC, c‐Myc, and HIF1‐α), metabolic product transport proteins (GLUTs), and glycolytic enzymes (HK2, PDK1, and LDH) [35]. The glucose transporter (GLUT) is responsible for the specialized uptake and transport of glucose [36]. In CRC, GLUT3 is highly expressed. Under conditions of limited glucose availability, GLUT3 accelerates glucose uptake and promotes nucleotide synthesis, thereby facilitating the growth of CRC cells through the AMPK/CREB1/GLUT3 axis. This effect is even more pronounced than the impact of GLUT1 on cellular growth [37, 38, 39]. In a low‐oxygen environment, hypoxia‐inducible factor 1 alpha (HIF1‐α) is activated, facilitating cellular metabolic adaptation. This adaptation includes an increase in glucose uptake and the acceleration of its conversion to lactate. Hexokinase 2 (HK2) plays a crucial role in glucose metabolism by catalyzing the conversion of glucose to glucose‐6‐phosphate. As a downstream target gene of HIF1‐α, HK2 is directly regulated. Elevated HK2 activity amplifies glucose absorption and metabolism within cells, thus providing the necessary energy and biosynthetic building blocks crucial for the growth and survival of tumor cells [40, 41]. Measures to counteract the overexpression or mutation of these factors, such as personalized epigenetic modulators, can potentially improve the unfavorable prognosis of patients with CRC.

The increased glycolysis rate caused by the Warburg effect promotes the production of reactive oxygen species (ROS). During glycolysis, some intermediate products may enter the mitochondria and participate in the electron transport chain, thereby increasing ROS production. ROS promote the tendency of cancer cells to utilize glycolysis for metabolism, thereby promoting the Warburg effect [42]. ROS can affect multiple signaling pathways, such as the PI3K/Akt and HIF‐1α pathways, which play important roles in regulating cellular metabolism. While modest levels of ROS disrupt signaling pathways that are advantageous for cell proliferation, an overabundance of ROS can inflict harm on nucleic acids and cell membranes, culminating in cell death. Cancer cells maintain appropriate levels of ROS through alterations in the mitochondrial redox potential, which is influenced by the Warburg effect. Furthermore, they enhance their antioxidant defense systems to offset excess ROS, thus promoting their proliferation and survival [43, 44]. Manganese superoxide dismutase (MnSOD/SOD2), a key mitochondrial enzyme, regulates the spectrum of ROS produced by organelles, significantly influencing cellular signaling dynamics. Studies have revealed that MnSOD upregulation perpetuates the Warburg effect by modulating mitochondrial ROS and AMPK‐mediated cancer signaling pathways. Curtailing MnSOD expression or inhibiting AMPK activity curbs cancer cell metabolism, underscoring the pivotal role of the MnSOD/AMPK axis in sustaining cancer cell bioenergetics. MnSOD has emerged as a potential biomarker for gauging cancer progression and is a central regulatory element in tumor cell metabolism [45, 46].

Compared with the complete oxidation of glucose, the glycolytic pathway generates fewer NADH molecules per unit of glucose consumed. This is because glycolysis produces only two NADH molecules per molecule of glucose, whereas the complete oxidation of a single glucose molecule can generate a greater number of NADH molecules. As a result, the overall production of NADH in tumor cells is reduced [47]. Additionally, under normal circumstances, NADH is reoxidized in the mitochondrial electron transport chain to NAD+, generating a large amount of ATP [48]. Therefore, compared with that in normal cells, the proportion of NADH that is reoxidized through the electron transport chain to produce ATP in tumor cells is decreased, and more NAD+ is regenerated through lactate fermentation [49]. In summary, the Warburg effect, by reducing the production of NADH and altering its utilization, significantly changes the internal energy metabolism of tumor cells, which is crucial for their growth and survival. Some researchers believe that in cancer cells, the Warburg effect occurs after the cycle and closure circuit between Glyceraldehyde‐3‐phosphate dehydrogenase (GAPDH) and LDH, leading to an increase in endogenous oxidative stress and carcinogenesis. Mitochondrial dysfunction in cancer cells results in high levels of glycolysis, which leads to the accumulation of NADH. H, thereby promoting the Warburg effect. Similarly, the oxidation of NADH. H produced excess NAD+ by LDH, which secondarily drives the GAPDH reaction to irreversibly produce NADH. Pyruvate acts as an antioxidant, whereas lactate acts as a pro‐oxidant, leading to an increase in endogenous oxidative stress and tumor hypoxia and high levels of glycolysis with NADH in cancer cells. On the basis of these findings, they proposed methods that might help prevent and combat the Warburg effect: the use of siRNAs to target GAPDH and LDH and the use of strong antioxidants to induce antioxidant–oxidant antagonism or antioxidant–salicylate antagonism to inhibit the Warburg effect, thus breaking the closed cycle [50].

Key enzymes involved in metabolic processes are our focal point in the study of metabolic reprogramming. Pyruvate kinase M2 (PKM2) is an isozyme of pyruvate kinase that activates the glycolytic pathway, enabling CRC cells to utilize glucose more efficiently for energy generation [51]. Studies have reported significantly elevated levels of PKM2 expression in CRC tissues compared with normal tissues. Furthermore, PKM2 contributes to the early diagnosis and prognosis assessment of CRC, with high expression associated with clinical features such as lymph node metastasis, tumor staging, and poor prognosis [52, 53, 54, 55]. PKM2 directly interacts with OTUB2, an OTU deubiquitinase overexpressed in CRC, inhibiting its ubiquitination by blocking the interaction between PKM2 and the ubiquitin E3 ligase Parkin. This process enhances PKM2 activity, consequently promoting glycolysis [56]. In the presence of fructose‐1,6‐bisphosphate (FBP), FOXM1D, an isoform of Forkhead box M1, binds to tetrameric PKM2, forming a hetero‐octameric structure. This interaction significantly limits the metabolic activity of PKM2, thereby promoting aerobic glycolysis [57]. These studies indicate that PKM2 has the potential to serve as a tumor marker for CRC, assisting in diagnosis, prognosis assessment, and monitoring treatment effectiveness. Figure 1 displays a portion of genes related to the Warburg effect and their interconnections in CRC.

As the fundamental metabolic process underlying the Warburg effect, subtle alterations in glycolysis have garnered significant attention in recent CRC research [58]. These studies often identify upstream factors associated with glycolytic enzymes and utilize cell line experiments or animal studies to elucidate their direct or indirect impacts on glycolysis in CRC cells. Table 1 summarizes the core molecules, research methods, and key conclusions of relevant studies.

**TABLE 1:** A compilation of reports on glycolysis abnormalities in CRC from the latest research.

Core molecule	Key downstream molecules	Impacts on glycolysis	Research method	Impacts on CRC	References
NSUN2	YBX1/m5C‐ENO1	↑	In vivo and vitro	Promote tumorigenesis and metastasis. Inhibit tumor immunity.	[59]
Plectalibertellenone A	TGF‐β/Smad pathway Wnt pathway	↓	In vitro	Inhibit proliferation, migration and invasion.	[60]
CMTM6	GLUT1	↑	In vivo and vitro	Promote proliferation and metastasis.	[61]
SHP2	PKM2	↑	In vivo and vitro	Promote proliferation, migration and invasion. Inhibit apoptosis and increase cisplatin resistance.	[62]
PTBP1	PKM2	↑	In vivo and vitro	Promote proliferation, invasion, migration.	[63]
TMPO‐AS1	PKM2	↑	In vitro	Promote proliferation and migration.	[64]
SPAG4	PI3K/Akt pathway	↑	In vitro	Promote growth and metastasis.	[65]
miR‐486‐5p	NEK2	↓	In vitro	Inhibit proliferation, migration, and cell stemness.	[66]
UBTD1	c‐Myc/HK2	↑	In vivo and vitro	Promote proliferation and migration.	[67]
BRIX1	GLUT1	↑	In vivo and vitro	Promote proliferation.	[68]
Tectorgenin	LncRNA CCAT2 miR‐145	↓	In vivo and vitro	Promote proliferation.	[69]
PHD2	NF‐κB pathway	↓	In vivo and vitro	Inhibit proliferation.	[70]
SELEENBP1	HIF1α	↓	In vivo and vitro	Inhibit proliferation and promote cell apoptosis.	[71]
GPR37	Hippo pathway	↑	In vivo and vitro	Promote metastasis.	[72]
AF9	PCK2	↓	In vivo and vitro	Inhibit proliferation and migration.	[73]
STING	HK2	↑	In vivo and vitro	Promote tumor immunity.	[74]
FABP4	ROS/ERK/mTOR pathway	↑	In vivo and vitro	Promote proliferation and stemness. Inhibit apoptosis.	[75]
CircVMP1	HKDC1	↑	In vivo and vitro	Promote proliferation and metastasis.	[76]
SETD8	HIF1α /HK2 pathway	↑	In vivo and vitro	Promote proliferation and inhibit cell apoptosis.	[40]
SOX2	GLUT1	↑	In vivo and vitro	Promote proliferation and migration. Promote vasculogenic mimicry.	[77]
PROX1	SIRT3	↑	In vivo and vitro	Promote proliferation.	[78]

### 2.2 Tricarboxylic Acid Cycle

The tricarboxylic acid (TCA) cycle is significantly affected during tumor development. In normal cells, the TCA cycle is one of the key metabolic pathways essential for cell survival, as it oxidizes organic substances into carbon dioxide and water, generating ATP for energy (Figure 1). However, in tumor cells, owing to factors such as hypoxia, nutrient deficiency, and increased metabolic demands, there is a preference toward anaerobic glycolysis, possibly inhibiting or altering the TCA cycle to some extent [79]. With the development of metabolomics, differences in the metabolic profiles between normal cells and tumor cells have increasingly been discovered. Some intermediate products of the TCA cycle, such as isocitrate and malate, are considered biomarkers for distinguishing the malignancy of tumors [80].

Genetic variations in the enzymes of the TCA cycle are significantly associated with CRC susceptibility. Single nucleotide polymorphisms (SNPs) in the TCA cycle are diagnostic biomarkers for CRC, and researchers have proposed identifying high‐risk CRC populations by detecting these SNPs [81]. OGT is the sole enzyme responsible for O‐GlcNAc glycosylation, and depleting OGT can significantly enhance TCA cycle metabolism in CRC cells. The OGT‐c‐Myc‐PDK2 axis is a key mechanism linking oncogene activation to the dysregulation of TCA cycle metabolism [82]. The TCA cycle is also associated with ferroptosis in tumor cells. Research has demonstrated that inhibiting activating transcription factor 3 (ATF3) and cystathionine β‐synthase (CBS) can target the mitochondrial cycle, increasing the susceptibility of CRC cells to ferroptosis. Therefore, blocking the ATF3‐CBS signaling axis may represent a potential therapeutic strategy for CRC [83].

In addition to ferroptosis, cuproptosis is a newly discovered type of programmed cell death related to cellular metabolism [84]. Cuproptosis participates in and accelerates the Fenton reaction in the body, generating highly reactive hydroxyl free radicals and ROS. Excessive ROS can damage cellular lipids, proteins, and DNA, ultimately leading to cell death. Additionally, the accumulation of ROS damages mitochondria by affecting their membrane potential, causing mitochondrial dysfunction. This process is a primary factor in how cuproptosis leads to the reprogramming of the TCA cycle [85] On the basis of the metabolic activity of the tumor, the degree of immune infiltration, and fibrosis, CRC can be divided into three distinct cuproptosis–hypoxia (CHS) subtypes. Studies indicate that in different CHS groups, patients exhibit significant differences in prognosis and sensitivity to conventional drugs [86]. In a study of CRC immunotherapy, researchers selected 10 genes associated with cuproptosis to determine the patterns of cuproptosis and related TME characteristics. They established the COPsig score to quantify the cuproptosis patterns in individual patients. Patients with higher COPsig scores were characterized by longer overall survival times, lower infiltration of immune and stromal cells, and a higher tumor mutation burden. Additionally, single‐cell transcriptomics analysis suggested that cuproptosis marker genes, such as ASPRV1 and TNKS, recruit tumor‐associated macrophages to the TME by regulating the TCA cycle and glutamine and fatty acid metabolism, thereby affecting the prognosis of CRC patients [87]. Cuproptosis‐related long non‐coding RNAs have also been demonstrated to be applicable in the chemotherapy and immunotherapy of CRC [88]. Moreover, there is a relationship between glycolysis levels and cuproptosis. 2‐DG, an inhibitor of glycolysis, can sensitize cells to cuproptosis. Additionally, galactose further promotes cuproptosis. The study also revealed that 4‐Octyl itaconate (4‐OI) significantly enhanced cuproptosis. Mechanistically, 4‐OI inhibits aerobic glycolysis by targeting GAPDH, a key enzyme in glycolysis, thereby sensitizing cells to cuproptosis [89]. This mechanism of promoting cuproptosis by inhibiting aerobic glycolysis is also a potential target for further research on targeted therapies.

### 2.3 OXPHOS

OXPHOS is closely linked to the TCA cycle. The TCA cycle generates NADH and FADH_2_ by oxidizing acetyl‐CoA, which is then used in OXPHOS to create a proton gradient across the inner mitochondrial membrane. This gradient drives ATP synthase to produce ATP, linking the TCA cycle's metabolites directly to OXPHOS's energy production. Owing to the occurrence of the Warburg effect and the suppression of the TCA cycle, the level of OXPHOS in tumor cells is reduced. Changes in the efficiency of OXPHOS can impact the energy balance and survival of cancer cells and may also encourage cancer cells to develop strategies to evade detection by the immune system, such as by altering the extracellular pH or affecting antigen expression [90]. Moreover, the reprogramming of OXPHOS can lead to stress in the intracellular environment, affecting cell signaling pathways, thus potentially affecting the proliferation and survival of cancer cells. However, OXPHOS has recently been identified as an emerging contributor to tumorigenesis and tumor progression. OXPHOS is important not only because of its ability to generate sufficient energy to support tumor cells but also because of its key role in regulating cell death and its ability to produce ROS, which can promote the malignant transformation of normal cells. Therefore, OXPHOS is involved in several aspects of cancer progression.

PPARγ coactivator 1α (PGC‐1α) profoundly influences OXPHOS by stimulating mitochondrial biogenesis, thereby causing cells to exhibit more oxidation and less glycolysis [91, 92]. Studies have shown that the expression of PGC‐1α is associated with tumor growth and metastasis. Moreover, the increase in OXPHOS mediated by PGC‐1α is a key factor leading to cancer resistance to first‐line chemotherapy drugs (oxaliplatin and 5‐FU) [93, 94, 95]. One study in which exosomes circ_0001610 from oxaliplatin‐resistant cell models were collected revealed that circ_0001610 upregulated the activity of PGC‐1α‐dependent OXPHOS, leading to reduced drug sensitivity in recipient cells [96]. Investigating the mechanisms of the exosome‐mediated increase in OXPHOS and exploring its link to chemotherapy resistance may provide a new target for CRC treatment.

OMA1 is a mitochondrial protease that is activated under stress conditions and promotes the development of CRC by driving metabolic reprogramming. The OMA1‐OPA1 axis is activated by hypoxia, increasing mitochondrial ROS to stabilize HIF‐1α, thereby promoting glycolysis in CRC cells. In contrast, under hypoxic conditions, depletion of OMA1 promotes the accumulation of components of the mitochondrial respiratory chain, such as NDUFB5 and COX4L1, supporting the suppression of OXPHOS in tumor cells. The results show that the coordinated action of OMA1 in glycolysis and OXPHOS promotes the development of CRC and highlight OMA1 as a potential target for the treatment of CRC [97]. In fact, many other factors affect OXPHOS in CRC. For example, leptin is a hormone secreted by adipose tissue. It can regulate fatty acid β‐oxidation and the TCA cycle in tumor cells through the c‐Myc/PGC‐1 pathway and greatly increase OXPHOS activity [98]. OXPHOS is weakened in CRC; however, even under these conditions, the OXPHOS process still promotes the growth and metastasis of CRC. Therefore, for tumor types such as CRC, which tend to rely on glycolysis, reducing OXPHOS might be more effective in limiting tumor development. However, many factors need to be considered in the treatment of tumors, and treatments that target metabolism should consider drug side effects and be personalized.

### 2.4 Other Glucose Metabolism Pathways

**FIGURE 2:**
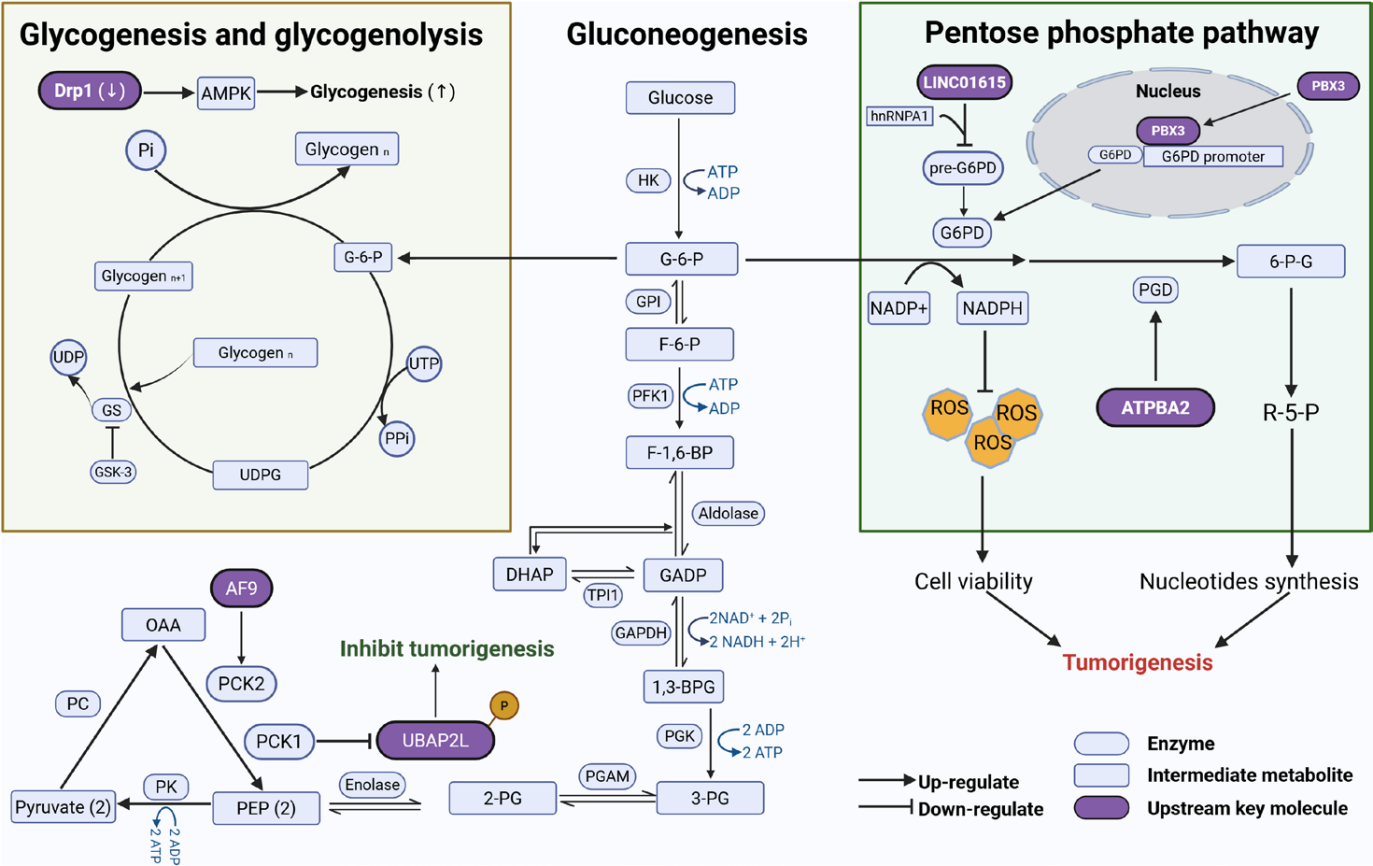
Illustration of the pentose phosphate pathway, gluconeogenesis, glycogenesis, and glycogenolysis in CRC, along with the genes and mechanisms associated with metabolic reprogramming displayed above.

The metabolic reprogramming of glucose metabolism in cells also involves several other aspects (Figure 2). The pentose phosphate pathway (PPP) is an important pathway in glucose metabolism that functions primarily to decompose glucose into pentoses while generating ATP and NADPH to provide the energy and materials required for cell growth and metabolism [99]. Owing to the need for metabolic activity that is greater than normal, the PPP often tends to increase in tumor cells [100]. Pre‐B‐cell leukemia transcription factor 3 (PBX3) belongs to the PBX family and is known for its elevated expression in various tumors, which is correlated with metabolic reprogramming. Glucose‐6‐phosphate dehydrogenase (G6PD) is a pivotal rate‐limiting enzyme in the PPP, and research has revealed that PBX3 functions as a positive regulator of G6PD. In CRC cells, PBX3 enhances the transcriptional activity of G6PD by directly binding to its promoter, thereby augmenting PPP activity. The PBX3/G6PD axis also has tumorigenic potential both in vitro and in vivo [101]. In addition, NADPH oxidase 4 (NOX4) enhances PPP activity through its interaction with G6PD, promoting the clearance of ROS and thereby protecting CRC cells from ferroptosis [102]. ATP13A2, a lysosome‐related transmembrane P5‐type ATP transportase, has been identified as a novel gene associated with the PPP. ATP13A2 primarily regulates the PPP by influencing the mRNA expression of phosphogluconate dehydrogenase (PGD). The overexpression of ATP13A2 promotes its nuclear localization by inhibiting the phosphorylation of TFEB, thereby increasing the transcription of PGD and ultimately affecting PPP activity. Conversely, a deficiency in ATP13A2 leads to reduced levels of PPP products and an increase in ROS, thereby inhibiting the growth and invasion of CRC [103].

Gluconeogenesis is a process opposite to glycolysis and involves the generation of glucose from noncarbohydrate precursors such as pyruvate, lactate, amino acids, and glycerol, which primarily occurs in the liver and, to a lesser extent, in the kidneys. This pathway plays a crucial role in maintaining normal blood glucose levels, particularly during extended periods of fasting or intense physical activity. Tumor growth requires the substantial synthesis of biomacromolecules, whereas gluconeogenesis consumes the potential carbon sources required for proliferation. Gluconeogenesis is an energy‐consuming process. Therefore, enhancing glycolysis and reducing gluconeogenesis are beneficial for tumor cells. Phosphoenolpyruvate carboxykinase (PCK) is a rate‐limiting enzyme in gluconeogenesis that catalyzes the conversion of oxaloacetate (OAA) to phosphoenolpyruvate (PEP) [104, 105]. PCK1 is the cytosolic isoform of the enzyme, and PCK1 deficiency can regulate gluconeogenesis and lead to inherited metabolic disorders [106]. Research has shown that the cytosolic level of PCK1 is significantly reduced in CRC, and high PCK1 deficiency can regulate gluconeogenesis and lead to inherited metabolic disorders. PCK1 counteracts CRC growth by inactivating the phosphorylation of UBAP2L at serine 454 and enhancing autophagy. On the other hand, from a metabolic standpoint, low PCK1 expression weakens gluconeogenesis, which to some extent benefits tumor growth [107].

Owing to their high energy demands, tumor cells break down glycogen more than they synthesize and store it. Glycogen synthase kinase‐3 (GSK‐3) is a serine/threonine kinase that does not directly catalyze glycogen synthesis. GSK‐3 indirectly regulates glycogen synthesis by inhibiting glycogen synthase (GS) activity. GSK‐3 phosphorylates GS, which reduces the activity of glycogen synthase, thereby inhibiting glycogen synthesis. GSK‐3 has been shown to be significantly correlated with tumor budding grade and PD‐L1 levels in CRC, providing new insights into immunotherapeutic approaches for patients with CRC who have a poor prognosis [108]. Dynamin‐related protein 1 (Drp1), a member of the dynamin family of GTPases, triggers the activation of a compensatory metabolic pathway through the activation of AMPK due to the silencing of Drp1 expression. In this pathway, increased glucose uptake funnels into the glycogen biosynthesis pathway, resulting in glycogen buildup in CRC cells. Research shows that targeting Drp1 for treatment is unlikely to be sufficient to eliminate cancer cells, but inhibiting the breakdown of glycogen could increase the chemosensitivity of cancer [109].

## 3 Dual Metabolic Reprogramming of Glucose and Lipid/Amino Acid in CRC

The metabolism of nutrients within the cell is a complex, dynamic, and interrelated process. In normal cells, the metabolism of the three major nutrients is closely interconnected and can be interconverted. In CRC cells, metabolic reprogramming is not limited to glucose metabolism; lipid metabolism and amino acid metabolism also undergo significant reprogramming [110]. The coordinated regulation of these metabolic pathways plays a crucial role in the progression and chemoresistance of CRC.

Short‐chain fatty acids (such as butyrate) can trigger a series of events in cells, inhibiting glycolysis in cancer cells and affecting fatty acid oxidation at the same time, thus exerting a dual influence on CRC metabolism through this metabolic chain reaction [111, 112, 113]. Recent reports have indicated that the combination of butyrate and selenite can inhibit colon cancer by influencing amino acid metabolism, which may serve as an effective strategy to increase the efficacy of chemotherapy in the future [114]. Silent information regulator of transcription, sirtuin 1 (SIRT1) is a NAD^+^‐dependent deacetylase that is well known for its impact on both glycolysis and lipid metabolism in tumors. SIRT1 exerts its effects by modulating the Wnt/β‐catenin pathway, thereby inhibiting glycolysis and promoting fatty acid oxidation, which in turn facilitates tumor cell proliferation. Additionally, the mutual negative feedback loop between Wnt and SIRT1 has recently emerged as a focal point of research [115, 116]. O‐GlcNAcylation is a type of post‐translational modification of proteins that involves the addition of N‐acetylglucosamine (GlcNAc) to serine or threonine residues of cytoplasmic and nuclear proteins [117]. Recent studies have shown that ACLY senses elevated glucose levels through O‐GlcNAc glycosylation, thereby enhancing lipid synthesis to promote the rapid proliferation of tumor cells [118]. Additionally, this O‐GlcNAc modification enhances the nuclear localization of SRPK2, regulates de novo lipid synthesis in tumor cells at the post‐transcriptional level, and consequently promotes tumor growth [119].

Recent studies have revealed that numerous molecules or signaling pathways can reprogram glucose metabolism in CRC, while simultaneously affecting the metabolism of various other substances (Table 1). In summary, the interplay among different metabolic reprogramming processes further promotes the progression of CRC through complex mechanisms. In recent years, research has increasingly focused on overall metabolic alterations in cancer. If their common upstream regulators could be inhibited, targeted metabolic therapies would achieve a significant improvement in efficiency (Table 2).

**TABLE 2:** Research progress on dual metabolic reprogramming in CRC.

Target molecule	Metabolic impact	Related downstream molecules or pathways	Main function	References
FUT2	Glucose and lipid	YAP/TAZ signaling SREBP‐1	Promote proliferation and metastasis.	[120]
SCD1	Glucose and lipid	—	Promote cancer development and progression.	[121]
PBX3	Glucose, lipid, and nucleotide	G6PD	Promote proliferation and inhibit intracellular ROS and apoptosis.	[101]
ATF4	Glucose and amino acid	SLC1A5	Promote viability, migration, invasion, and metastasis.	[122]
GLUT5	Glucose and lipid	AKT1/3‐miR‐125b‐5p	Induce migratory activity and drug resistance.	[123]
GLUT14	Glucose and creatine	HIF1α	Promote proliferation.	[124]
ARL15	Glucose & lipid	AKT/AMPK	Promote the occurrence of colon cancer.	[125]
CRMP2	Glucose & lipid	GLUT4	Regulate apoptosis/proliferation, cell migration and differentiation.	[126]
Maggot extracts	Glucose & lipid	HMOX1/GPX4 signaling pathway	Inhibit proliferation and induce ferroptosis.	[127]
PEPCK	Glucose & amino acid	mTORC1	Promote proliferation.	[105]
ChREBP	Glucose & lipid	p53 pathway	Regulate proliferation and cell cycle arrest.	[128]
SIRT6	Glucose & lipid	PKCζ	Regulate lipid homeostasis.	[129]
RARRES1	Glucose & lipid	PPAR pathway	Regulate fatty acid metabolism in epithelial cells.	[130]
PLOD2	Glucose & amino acid	STAT3 pathway；HK2	Promote proliferation and invasiveness.	[131]
ADMA	Glucose & lipid, amino acid & nucleic acid	—	Regulate apoptosis and cell cycle.	[132]
RPIA	glucose & nucleic acid	CARM1	Promote ROS clearance and growth.	[133]
ART1	Glucose & amino acid	PI3K/AKT/HIF1α	Promote proliferation.	[134]
AMPK	Glucose & amino acid	PPARδ	Inhibit colon tumor growth.	[135]
c‐Src	Glucose & amino acid	PFKFB3	Promote tumorigenesis and progression.	[136]
PFKFB4	Glucose & amino acid	—	Regulate immune infiltration.	[137]
LINC01764	Glucose & amino acid	c‐Myc	Promote proliferation, metastasis, and 5‐FU resistance.	[138]
LINC01615	Glucose & lipid & nucleotide	G6PD	Promote proliferation and inhibit intracellular ROS and apoptosis.	[139]

## 4 Therapeutics Targeting Glucose Metabolic Reprogramming

Targeting genes and proteins associated with glucose metabolic reprogramming can effectively inhibit the energy supply to tumor cells, disrupt key metabolic pathways, and utilize specific metabolic characteristics for targeted treatments [116, 140]. This approach holds significant promise and offers new insights and strategies for the treatment of CRC. This section introduces some of the latest research developments on the reprogramming of glucose metabolism in CRC cells.

Exogenous drugs or reagents have been employed in research on glucose metabolism. A class of drugs called mTOR inhibitors targets the rapamycin signaling pathway, effectively suppressing glucose metabolism in CRC cells. Inhibition of LDHA can sensitize CRC cells to rapamycin, and the combined use of rapamycin with the glycolysis inhibitor oxamate exhibits a potent inhibitory effect on CRC [141]. Xanthohumol (XN) is a prenylated flavonoid compound that is obtained primarily through extraction from hops. Low doses of XN can inhibit the proliferation and progression of HT29 cells by downregulating inflammatory signaling and cellular metabolism. Specifically, low doses of XN can suppress glucose and oxidative metabolism as well as lipid peroxidation with minimal effects on cell viability. Although researchers have indicated that strategies are being developed to increase the bioavailability of XN, this study has not yet been completed in terms of metabolic transformations [142].

2‐DG can enter the glycolytic metabolic pathway in cells and competitively inhibit the phosphorylation of glucose to glucose‐6‐phosphate, greatly reducing energy production. 2‐DG has a broad range of metabolic effects, including the depletion of cellular energy, intensification of oxidative stress, and interference with N‐linked glycosylation. It also activates various signaling pathways, such as the PI3K/AKT, MAPK, and AMPK pathways [143, 144, 145]. These events are interconnected to some extent and are collectively influential. The toxicity observed after treatment with 2‐DG was caused by one or more of the mechanisms mentioned above. Studies have shown that treating CRC cells resistant to chemotherapeutic drugs, such as 5‐fluorouracil or oxaliplatin, with 2‐DG can inhibit glycolytic enzymes in cells, reduce the invasive capability of tumor cells, and improve their resistance to drugs [146, 147, 148]. Nevertheless, the therapeutic efficacy of 2‐DG is limited by high‐dose systemic toxicity, and research into its resistance mechanisms has attracted attention. Recent studies have shown that 2‐DG inhibits glycolysis, which in turn increases the expression of thioredoxin‐1 (Trx‐1) in CRC cells. Trx‐1 overexpression can reduce the cytotoxicity of 2‐DG. Mechanistically, Trx‐1 increases glutathione (GSH) levels by modulating SLC1A5, thereby rescuing the cytotoxic effects of 2‐DG on CRC cells [149]. Combining glycolysis inhibition with Trx‐1 or SLC1A5 inhibition may be a promising strategy for the treatment of CRC.

Theoretically, endogenous molecules that are capable of inhibiting glucose metabolism are the best natural therapeutic agents. The active form of vitamin D, calcitriol, can inhibit glycolysis and tumor growth in human CRC cells [150]. Fructose‐2,6‐bisphosphatase 3 (PFKFB3) catalyzes the production of fructose‐2,6‐bisphosphate and acts as an oncogene, and its mechanism is linked to the expression of IL‐1β and TNF‐α. Inhibitors of PFKFB3 and PFK158 can reduce the survival rate, migration, and invasion of CRC cells caused by the overexpression of PFKFB3 [151]. Vitamin C induces ATP depletion and a decrease in the phosphorylation of pyruvate dehydrogenase E1‐α at serine 293, thereby increasing the activity of pyruvate dehydrogenase (PDH) and citrate synthase. Furthermore, vitamin C downregulated pyruvate dehydrogenase kinase‐1 (PDK‐1) in KRAS‐mutant CRC cells through prolyl hydroxylation (Pro402) of HIF‐1α, significantly affecting the TCA cycle and mitochondrial metabolism in multiple ways. Vitamin C may play a role in the clinical treatment of anti‐EGFR chemotherapy‐resistant CRC [152, 153]. In summary, endogenous glucose metabolism inhibitors not only directly suppress tumor cell growth but also increase the sensitivity of various tumors to chemotherapeutic drugs. Glycolysis inhibitors are targeted metabolic drugs that are closest to widespread clinical use, bringing hope to patients with advanced CRC.

Immune cells in CRC also undergo metabolic reprogramming. Using specific methods to help them adapt to the TME and function more effectively is also a therapeutic strategy. A P2A‐linked bicistronic vector named GT5‐19BBz was constructed to express GLUT5 and an anti‐CD19 CAR. GLUT5‐engineered CAR‐T cells more effectively utilize fructose in glucose‐restricted tumor environments, thereby increasing the activity of the TCA cycle and the pentose phosphate pathway. In a melanoma mouse model, GLUT5‐engineered T cells accumulated in the TME and inhibited tumor growth. Moreover, combination therapy with PD‐1/CTLA‐4 immune checkpoint inhibitors significantly improved the survival rate of tumor‐bearing mice [15]. In addition, studies targeting dendritic cells (DCs) have shown that NCoR1 mediates the regulation of glycolysis and fatty acid oxidation in DC cells, which is beneficial for their immune tolerance [154]. Combining immunotherapy with targeted metabolism can overcome the immunosuppressive microenvironment, enhance the function of immune cells, and improve the response rate of immunotherapy through multitarget collaboration. This approach is not only a hot topic of research in recent years, but is also set to become a new strategy for future cancer treatment.

Clinical studies have applied glucose metabolic reprogramming‐related genes as diagnostic and prognostic markers for CRC (Table 3). (https://clinicaltrials.gov/) Some of these studies have been completed, but many are still in the recruitment phase. Metabolic imaging techniques such as FDG‐PET have been employed in many clinical trials. FDG‐PET is a molecular imaging technology that combines positron emission tomography (PET) and computed tomography (CT). It can reflect the metabolic activity of tumor cells by detecting their glucose uptake. By analyzing the distribution of FDG uptake, researchers can better understand the metabolic characteristics of CRC and provide a basis for personalized treatment.

**TABLE 3:** Clinical trials on metabolic alterations in CRC.

NCT number	Official title	Study status	Conditions	Interventions	Primary outcome measures	Enrollment	Study type	Application
NCT04516681	Vitamin C intravenously with chemotherapy and Adebrelimab in metastatic colorectal cancer with high expresison level of GLUT3.	Recruiting	Colorectal Cancer Vitamin C GLUT3	Ascorbic acid FOLFOX protocol Adebrelimab	Target response rate, which refers to the total tumor response rate of subjects receiving combination therapy of ascorbic acid and FOLFOX RI+/− bevacizumab versus receiving the latter alone.	400	Interventional	Treatment
NCT01591590	Correlating the tumoral metabolic progression index measured by serial FDG‐PET CT and apparent diffusion coefficient measured by MRI to patient's outcome in advanced colorectal cancer.	Completed	Colorectal Cancer	FDG PET‐CT MRI	Mortality, 12 months.	53	Interventional	Diagnosis
NCT00184782	Exploratory study on FDG‐PET scanning in evaluating the metabolic activity of liver metastases before and after resection of the primary tumor in patients with a colorectal malignancy.	Unknown	Colorectal Neoplasms	FDG‐PET	Increase in metabolic activity of liver metastases after resection of the primary tumor compared to the activity of metastases in patients without primary tumor resection.	30	Interventional	Diagnosis
NCT06011473	Continuous glucose monitoring for colorectal cancer.	Recruiting	Continuous Glucose Monitoring Colorectal Cancer	CGM (FreeStyle Libre 3)	Feasibility of CGM system.	40	Interventional	Supportive care
NCT02700555	Surveillance of metabolic parameters in patients who will receive chemotherapy after surgical resection of colorectal cancer: KBSMC colon cancer cohort.	Terminated	Colorectal Cancer	—	Incidence of newly developed diabetes mellitus in patients with colorectal cancer after post‐operative chemotherapy, up to 12 months.	23	Observational	Supportive care
NCT06614660	Multimodality metabolism and imaging genomics model for predicting the short‐term and long‐term outcomes for colorectal cancer patients.	Active Not recruiting	Colorectal Cancer	Metabolism genomics and imaging genomics	Overall survival, assessed up to 60 months.	800	Observational	Prognosis biomarker
NCT06117241	A transdisciplinary approach to investigating metabolic and risk of early‐onset colorectal cancer: a randomized intervention trial in human dyads and mechanistic study in animals.	Recruiting	Colorectal Cancer	Imagine healthy	Blood, TNFα, IL‐6, CRP, reversal of metabolic dysfunction, Baseline, 3‐month, 6‐month.	180	Interventional	Prevention
NCT01426490	The effects of vitamin B‐6 status on homocysteine, oxidative stress, one‐carbon metabolism and methylation: cross‐section, case–control, intervention and follow‐up studies in colorectal cancer.	Unknown	Colorectal Cancer	Dietary supplement	The oxidative stress (TBARs), antioxidant activities and DNA methylation in colorectal cancer patients, 12 months.	300	Intervention	Prevention
NCT06643429	Impact of environmental factors and metabolomics on colorectal cancer development and prognosis based on environmental factors and metabolomics.	Active Not recruiting	Colorectal Cancer Metabolic Disease	Diagnostic test: colorectal cancer	Rate of postoperative complications, assessed up to 2 months after surgery.	700	Observational	Diagnosis
NCT00394615	Metabolic imaging: predicting histopathologic response to preoperative chemoradiotherapy for locally advanced rectal cancer.	Terminated	Rectal Cancer	Metabolic imaging	To learn whether new medical imaging technology can help predict the response of rectal cancer to preoperative chemotherapy and radiation therapy.	15	Intervention	Treatment

Changes in enzyme activity resulting from metabolic reprogramming are key factors in the pathogenesis of CRC and in its adaptive stress response. Modulation of these metabolic enzymes significantly improves treatment outcomes and survival rates in patients with cancer. Extensive research has confirmed the viability of targeting the key genes and proteins involved in the reprogramming of glucose metabolism. Combining targeted therapy with other treatments has a positive impact on limiting malignant tumors and contributes to improving patient prognosis and survival rates.

## 5 Conclusion and Perspective

Metabolic reprogramming is an intrinsic mechanism in tumor cells, and almost all types of tumors exhibit several common metabolic alterations, especially alterations in glucose metabolism. CRC cells consume a substantial amount of glucose and produce lactate via the Warburg effect to meet their continuous energy demands. Increased lactate production also contributes to immunosuppression. In CRC, the tricarboxylic acid cycle is downregulated and associated with cellular cuproptosis (Figure 1). The levels of OXPHOS are also reduced, affecting multiple signaling pathways and immunotherapy in CRC cells. Upregulation of the pentose phosphate pathway generates reducing equivalents to counteract the oxidative stress encountered by rapidly proliferating cancer cells. Gluconeogenesis and glycogen metabolism also exhibit a certain degree of reprogramming to meet high energy and biosynthesis demands (Figure 2).

Similar to other malignant tumors, the development and progression of CRC necessitate metabolic activities distinct from those of normal cells. CRC cells undergo metabolic reprogramming of glucose to acquire the additional energy necessary for their growth and proliferation, establishing a TME conducive to invasion and metastasis, thus facilitating the rapid proliferation of the cell population. In recent years, further insights into the molecular mechanisms underlying the development and progression of CRC have emerged, particularly into the pathways associated with tumor growth metabolism. Glucose metabolic reprogramming also affects the treatment of CRC, with therapies targeting metabolism having significant research potential. Inhibiting key metabolic pathways not only slows the rate of tumor cell proliferation and spread but also increases resistance to chemotherapy and enhances the efficacy of immunotherapy [155].

**FIGURE 3:**
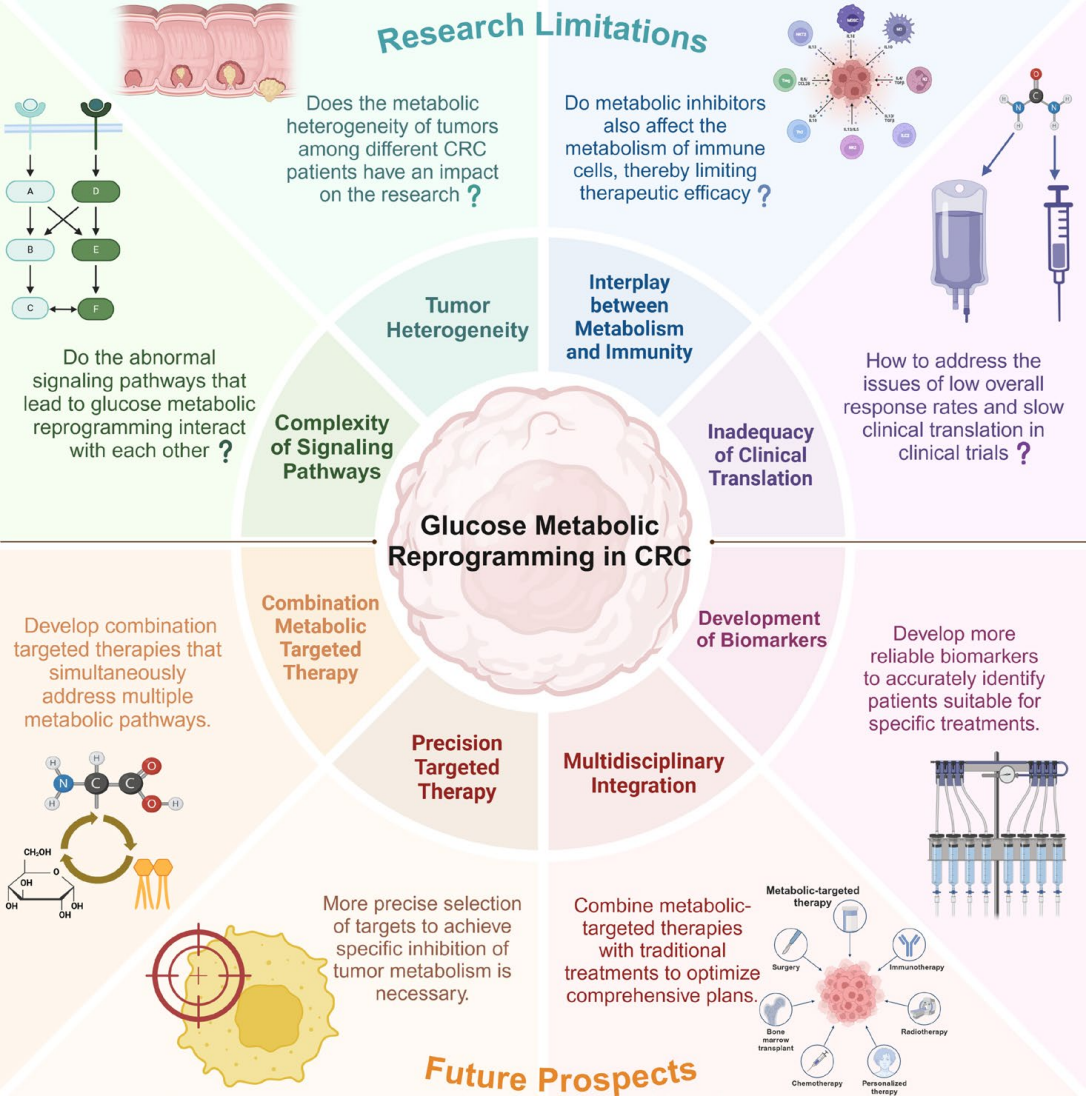
The current challenges in basic and clinical research on glucose metabolic reprogramming in CRC, as well as the key issues that future research should prioritize.

Despite the many advances in the study of CRC metabolomics, research on the interrelationships between the various signaling pathways that affect glucose metabolism in CRC remains incomplete. Moreover, the impact of metabolic reprogramming on the TME and tumor immunity is a valuable research direction, but there is currently a scarcity of clinical translation in this area [156, 157]. Under physiological conditions, the metabolism of different nutrients is interconnected and can be interconverted, a phenomenon that is even more pronounced in tumors. In recent years, an increasing number of studies have revealed the interactions between glucose metabolism and lipid or amino acid metabolism. Future research should continue to focus on precise biomarkers and targeting points that can simultaneously modulate the metabolism of multiple substances. Owing to insufficient validation in clinical trials, uncertainties and limitations exist in the treatment of targeted metabolism. These potential limitations include effects on the metabolism and biosynthesis of normal cells, which could lead to adverse reactions and side effects in patients. Considering that such treatment may require a sustained period to achieve efficacy, long‐term therapy could result in increased drug tolerance and treatment costs for patients. Additionally, more thought should be given to how to integrate metabolism‐targeting therapies with traditional cancer treatments to devise better comprehensive treatment plans for CRC patients. In summary, although there are numerous studies on glucose metabolism reprogramming and its clinical applications, many challenges and issues remain to be addressed (Figure 3).

As genomics, energy metabolism, and other theories and technologies have advanced, we anticipate uncovering more CRC‐related metabolic characteristics. This progress will enable the development of efficient and safe targeted drugs for clinical application. In summary, by deepening our understanding of the metabolic mechanisms of glucose, we aim to prevent, diagnose, and treat CRC from a metabolic perspective, thereby further improving the cure rate and survival time of patients with CRC.

## Author Contributions

R.Z. organized the literature and drafted the manuscript. F.W. and J.W. made the mechanism diagram. X.Z. and Y.W. provided critical comments and supervised this study. All authors contributed to the writing, review, and editing of the original draft and validated the final manuscript.

## Disclosure

Search Strategy and Selection Criteria: We searched PubMed for review articles published in English between January 1, 2015, and April 26, 2024, which included “Colorectal Neoplasms” with “Metabolic Reprogramming” OR “Glucose Metabolism Disorders” OR “The Warburg Effect” OR “Targeted Therapy.” We also searched the reference lists of the articles identified by this search strategy and selected those that were judged to be relevant. Review articles provide readers with an overview of some aspects of glucose metabolism mechanisms. As this article presents the discovery and historical evolution of the Warburg effect theory, it cites a small number of older publications.

## Conflicts of Interest

The authors declare no conflicts of interest.

## Data Availability

Data sharing not applicable to this article as no datasets were generated or analyzed during the current study.
